# Measuring the density of iodine depositions: Detecting an invisible residual tumor after conventional transarterial chemoembolization

**DOI:** 10.1371/journal.pone.0227972

**Published:** 2020-01-29

**Authors:** Johannes Haubold, Johannes M. Ludwig, Yan Li, Matthias Buechter, Axel Wetter, Lale Umutlu, Jens M. Theysohn

**Affiliations:** 1 Department of Diagnostic and Interventional Radiology and Neuroradiology, University Hospital Essen, Essen, Germany; 2 Department of Gastroenterology and Hepatology, University Hospital Essen, University Duisburg-Essen, Essen, Germany; Chang Gung Memorial Hospital at Linkou, TAIWAN

## Abstract

**Purpose:**

The purpose of this study is to evaluate the use of density measurements in the diagnosis of an underlying residual tumor beyond iodine depositions after Lipiodol-based conventional transarterial chemoembolization (cTACE).

**Method and materials:**

Thirty follow-up CT scans of 20 patients 6–12 weeks after Lipiodol-based cTACE, receiving a digital subtraction angiography at the same time, were analyzed. Reference for the detection of a residual tumor was the angiography, and a visible contrast enhancement was categorized as a residual tumor (*n* = 16 with residual tumor; *n* = 14 without residual tumor). The density of the iodine depositions was measured in all containing slices in non-contrast-, arterial- and portal venous-phase CT scans, with a slice thickness of 5.00 mm. The mean density of the iodine deposition during the portal venous phase was subtracted from the mean density of the arterial phase to calculate the density changes (a positive enhancement score represents washout in the portal venous phase). In addition, a quotient relating to the non-contrast measurement was evaluated.

**Results:**

Patients with a residual tumor displayed significantly higher enhancement scores in favor of density reduction between the arterial and portal venous phases, compared to patients without a residual tumor (1.41 ± 3.59, *n* = 14 vs. -13.97 ± 2.88, *n* = 16; *p*-value < 0.01). Furthermore, 87.75% of patients with an enhancement score higher than -1.00 (*n* = 9) had a residual tumor, whereas 100.00% of patients with an enhancement score lower than -20.00 (*n* = 6) were shown to be tumor-free. The enhancement score quotient resulted in similar findings.

**Conclusion:**

After cTACE in patients with hepatocellular carcinoma (HCC), the presence of a viable tumor correlated with enhancement scores based on the density measurements of iodine depositions in different phases of the CT scan. Low enhancement scores were associated with completely treated tumors and can aid the decision process to avoid possibly unnecessary angiographies.

## Introduction

In 2018, hepatocellular carcinoma (HCC) was the fifth most common cause of cancer in men and the second most common cause of cancer-related death worldwide [1]. It is associated with liver cirrhosis, Hepatitis B/C/D, alcohol abuse and obesity, reducing the operability and therapeutic options [2]. This leads to a poor median overall survival of 20 months after diagnosis [3].

Curative options include liver resection and radiofrequency ablation (RFA). While these options are only viable in limited diseases, transarterial chemoembolization (TACE) is a safe and efficient therapeutic option for an unresectable HCC [4]. Different TACE techniques are available: The initial TACE, also called conventional TACE (cTACE), was invented using Lipiodol combined with chemotherapeutics in the early 1980s. Whether other TACE techniques, such as drug-eluting bead TACE (DEB-TACE), are superior to Lipiodol-based TACE in overall survival has not yet been demonstrated [5]. Thus, cTACE is still the standard of care.

Some studies indicate a higher rate of postinterventional pain after DEB-TACE [6] as well as a higher induction of biliary and global hepatic damages [7], whereas other studies suggest a better toleration and a shorter hospital stay after DEB-TACE compared to cTACE [8].

At the same time, the radiopacity of Lipiodol, which was historically used as a contrast agent, helps to monitor the treatment. Incomplete deposition inside HCC or washout of Lipiodol can be a marker for a tumor relapse. Moreover Chen et al. have demonstrated that “good Lipiodol depositions” vs. “poor Lipiodol depositions” correlate with the risk of death and progression-free survival after Lipiodol-based TACE in patients with unresectable HCC [9].

In contrast, the opacity of iodine depositions can hide hyperarterialization or contrast washout, thereby making the diagnosis of a residual tumor difficult.

With regard to other imaging techniques, in magnetic resonance imaging (MRI), hypervascularization can be assessed more efficiently compared to computed tomography [10], whereas iodine depositions could not be evaluated in MRI. Those imaging difficulties in MRI and CT imaging techniques lead to repeated digital subtraction angiographies (DSAs) as a reference standard in the follow-up after Lipiodol-based TACE. The aim of this retrospective study was to evaluate the use of density measurements after Lipiodol-based cTACE in the diagnosis of a residual tumor. A technique indicating tumor control by measuring the contrast enhancement inside of iodine depositions in computed tomography could avoid unnecessary diagnostic digital subtraction angiographies.

## Material and methods

### Study design

We retrospectively evaluated the computed tomographies (CTs) between 01/2015 and 01/2018 of patients with unresectable HCC after Lipiodol-based cTACE. The patients were examined on a SOMATOM Definition Flash, SOMATOM Force or SOMATOM Definition AS CT scanner (Siemens Healthcare, Erlangen, Germany). Digital subtraction angiography (DSA) was performed up to one months after the CT on a Toshiba Infinix DP-i (Toshiba Medical Systems, Tokio, Japan) or Philips Allura^™^ (Philips Healthcare, Best, Netherlands) system. The DSA was used as a reference standard to define patient groups: Patients with a contrast enhancement inside or next to the iodine depositions in the angiography were categorized as having a residual tumor, and patients without contrast enhancement were categorized as being without a residual tumor.

### Ethics statement

This study was conducted in accordance with all guidelines set forth by the approving institutional review board of the University Hospital Essen–Approval number: 17-7540-BO. Written informed consent was waived by the Institutional Review Board because of the retrospective nature of the study. Moreover, all data were completely anonymized before they were included in the study.

### Measurement

After cTACE, iodine depositions (remaining Lipiodol with a high density) can hide an underlying residual tumor or fibrous tissue. In theory, fibrous tissue should depict a slowly progressing contrast enhancement from the arterial to the venous phase because of a diffusion-based contrast enhancement. In contrast to that, an underlying HCC should display a fast contrast enhancement in the arterial phase, followed by a fast contrast washout in the venous phase as a result of hypervascularization of the tumor [11].

**Fig 1:**
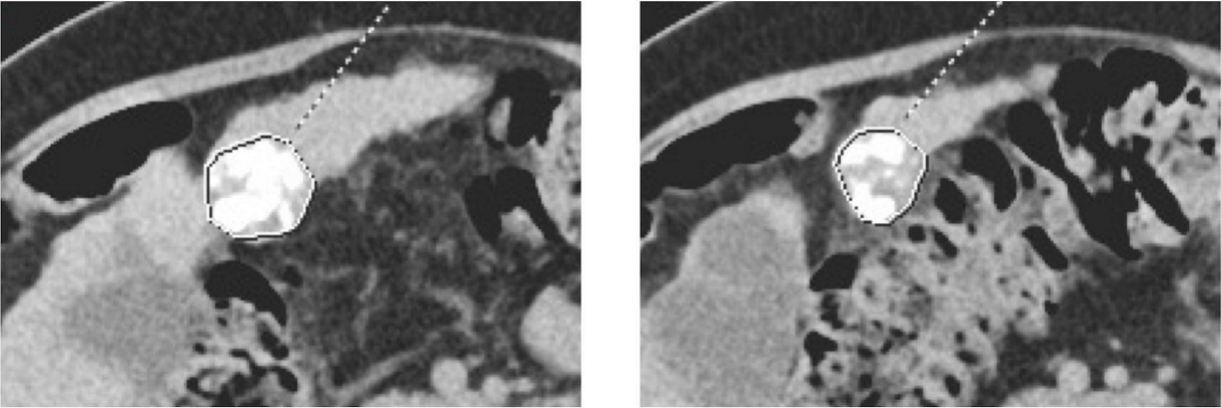
Example measurement. Measurement of the density and area of iodine depositions using a polygon ROI in Syngo.via. The polygon ROI was plotted around the iodine depositions of HCC, previously treated with cTACE in a superselective manner.

To quantify the contrast behavior of the underlying tissue beyond the iodine deposition, density and area measurements of iodine depositions were made in non-contrast, arterial and portal venous contrast images (5-mm slice thickness). Therefore, a polygon region of interest (ROI) was plotted around the iodine depositions on every slice, measuring the mean density and the area, as illustrated in Fig 1. The density measurements were taken using the Syngo.via (Siemens Healthcare, Erlangen, Germany). Thereafter, we multiplied the mean density of iodine depositions on every slice with the area of the iodine depositions and cumulated them to quantify the total density and volume of the iodine deposition. Afterward, we divided the density through the volume to analyze the relative density of the iodine depositions.

In the next step, we subtracted the relative density of the iodine deposition in the venous phase from that of the same deposition in the arterial phase to visualize the contrast behavior in the following equation, referred to as the “enhancement score.”

**enhancement score = arterial density–venous density**

**Fig 2:**
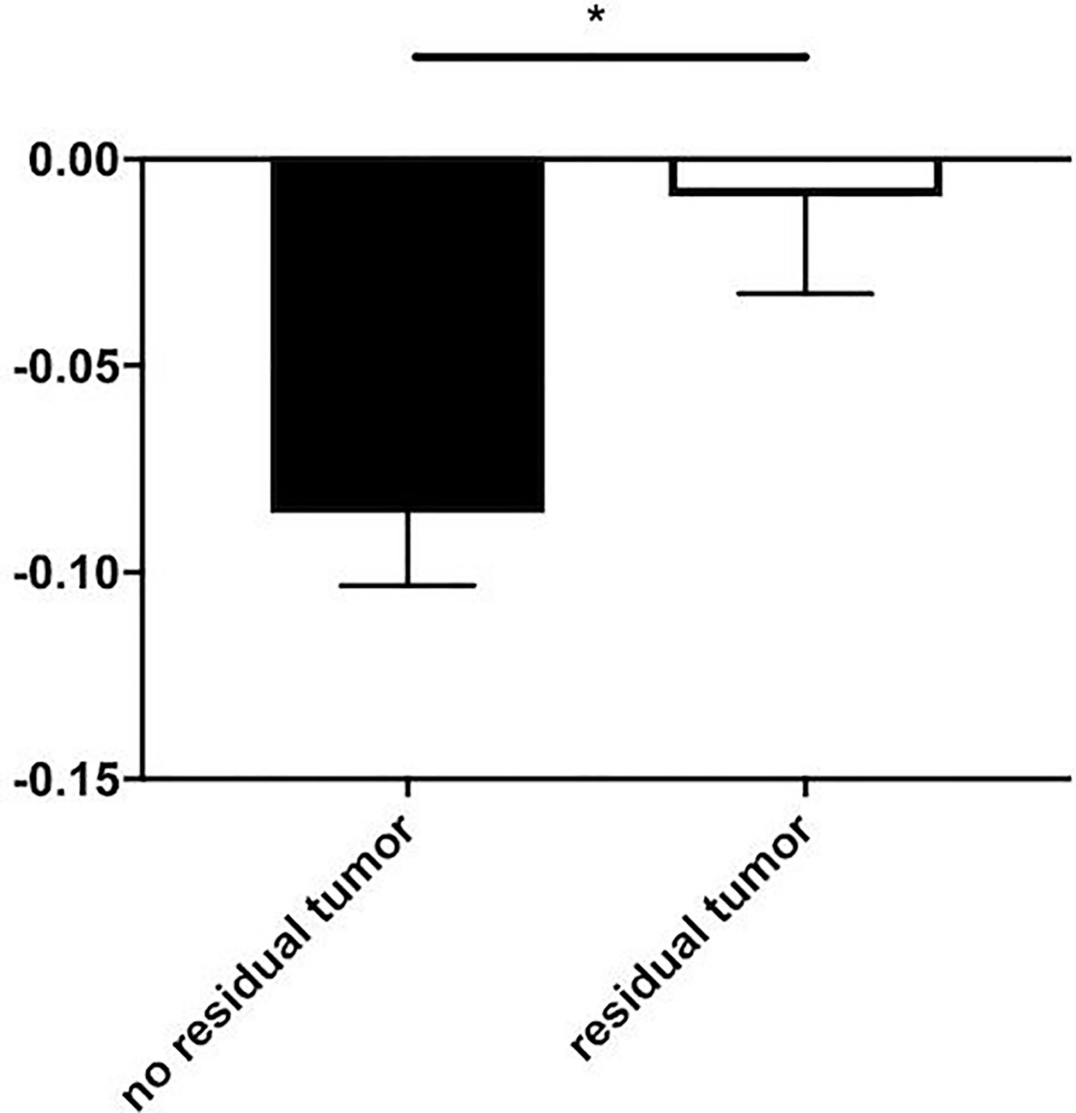
Comparison of enhancement scores relative to non-contrast densities (mean+SEM). Relative enhancement scores (enhancement score / non-contrast density) in patients with three-phasic computed tomographies (n = 25) did not lead to a better differentiation between patients with and without a residual tumor (-0.09 ± 0.02, n = 14 and -0.01 ± 0.02, n = 11; p < 0.05).

To furthermore test whether relativization onto the non-contrast density could be beneficial, we divided the enhancement score through the mean density of iodine deposition in the non-contrast phase, as illustrated in Fig 2.

As a result of this, residual HCC should display a higher score after subtracting the portal venous mean density from the arterial mean density, whereas slow but constant contrast accumulation should exhibit lower scores in patients with completely treated tumors.

### Baseline characteristics of patients

All patients had either single HCC or a single recurrence of a previously multifocal HCC without vascular invasion. Therefore, all patients were BCLC A with single HCC or B with focal singular recurrence, which was treated in a superselective manner with cTACE. Only patients with no residual vital tumor visible in computer tomography were included; therefore, no tumors with ill-defined margins were included. The cTACE was performed only in patients with adequate liver function with a bilirubin value below 2.00 mg/dl. The therapy decision for cTACE was made in a multidisciplinary tumor conference for all patients. The baseline characteristics of all patients are presented in Table 1.

**Table 1:** Baseline characteristics of all patients.

Patient	Diameter (mm)	Distribution	Segment	BCLC	Child-Pugh	Bilirubin (mg/dl)
1.	25	multifocal	S4a	B	A	1.00
2.	20	multifocal	S4a	B	A	0.50
3.	31	singular	S2/3	A	A	0.80
4.	34	multifocal	S4a	B	A	0.90
5.	43	singular	S7/8	A	A	1.50
6.	27	singular	S4b	A	A	0.40
7.	23	singular	S6	A	A	1.20
8.	23	singular	S7/8	A	A	0.70
9.	19	singular	S4b	A	A	0.60
10.	13	singular	S6	A	A	0.90
11.	47	singular	S6/7	A	A	0.80
12.	24	singular	S5	A	A	0.70
13.	34	singular	S2	A	A	0.90
14.	52	singular	S7/8	A	A	1.70
15.	17	singular	S3	A	A	0.70
16.	23	singular	S2	A	A	0.90
17.	28	singular	S3	A	A	1.20
18.	43	multifocal	S2/3	B	A	1.10
19.	17	singular	S5/6	A	A	1.30
20.	45	singular	S7	A	A	1.20

### Diagnostic DSA and conventional TACE procedure

With the Seldinger technique, the right common femoral artery was punctured under local anesthesia, and a sheath introducer was inserted to secure the access point. Selective hepatic angiography was then performed with a diagnostic catheter. The tip of the catheter was placed in the truncus coeliacus, the superior mesenteric artery, the common hepatic artery or the proper hepatic artery to identify the vascular supply of the liver. In a subsequent diagnostic liver angiography, the tumor recurrences were identified.

The cTACE was performed by a consultant interventional radiologist with a 3-French microcatheter (Renegade, Boston Scientific, Natick, MA, USA). Tumor feeders were evaluated in the DSA with iodine-based intravenous contrast media and embolized on a subsegmental branch level with an emulsion containing 75.00 vol% Lipiodol and 25.00 vol% Mitomycin dissolved in contrast media. The emulsion was administered in 1-ml steps until stasis or until reaching a maximum dose of 15.00 ml. Afterwards, the vessel was occluded with gelfoam. To repeat cTACE, the same vessel was used if reperfused, or newly visible feeding arteries were utilized if they were not visible before. Therefore, the feeding vessel status was peri-interventionally evaluated using Cone-beam CT.

As premedication, all patients received peri-interventional intravenous Kevatril (3.00 mg), Novalgin (2.50 g) and Dipidolor (7.50 mg) to avoid possible side effects such as nausea and pain.

### Computed tomography

Computed tomographies with iodine-based intravenous contrast media were performed on a SOMATOM Definition Flash, SOMATOM Force or SOMATOM Definition AS CT scanner (Siemens Healthcare, Erlangen, Germany). Reconstructions with a layer distance and thickness of 5-mm were used to evaluate the iodine deposition in the non-contrast, arterial and portal venous contrast phases.

The parameters were as follows: 0.50-s rotation time, a pitch of 0.60 and 0.60-mm detector collimation. The tube voltage was automatically adjusted to each patient using CAREkV (Siemens Healthcare, Erlangen, Germany) with a reference of 100.00 kV. In addition, CareDose 4D was used for an automatic tube current modulation adapted to patient anatomy for effective mAs (Siemens Healthcare, Erlangen, Germany), with a quality reference of 240.00 mAs. The non-contrast scan was acquired without a delay. Furthermore, bolus tracking with an ROI placed in the abdominal aorta was used to acquire the arterial phase, while the portal venous phase was acquired with a delay of 50.00 s after contrast media injection. Contrast media (1.50 ml/kg) were injected at a flow rate of 2.50–4.50 ml/s. The images were reconstructed using the sinogram affirmed iterative reconstruction (SAFIRE) technique.

### Statistical analysis

The distribution normality of the enhancement scores was analyzed with the Shapiro-Wilk test showing a Gaussian distribution. The enhancement scores of patients with a residual tumor were consequently compared to patients without a residual tumor using Student’s *t*-test. The measured Lipiodol volumes in patients with and without residual tumors, in the arterial and venous phases, were compared using a one way ANOVA and the Bonferroni post-test. Scores with a *p*-value of less than 0.05 were considered statistically significant. Furthermore, the *p*-value of receiver operating characteristic (ROC) curves was calculated and compared to a ROC curve with an AUC of 0.50. All statistics were performed using GraphPad Prism 7 (GraphPad Software, San Diego, USA).

## Results

Thirty computed tomographies from 20 patients (15 men and five women) were enrolled in our study: 25 computed tomographies with three phases (non-contrast, arterial and portal venous phases; 14 with a residual tumor and 11 without a residual tumor) and five computed tomographies with two phases (arterial and portal venous phases; three with a residual tumor and two without a residual tumor). Later, five computed tomographies with only two acquired phases were included after a subanalysis of the first 25 did not yield additional information using the non-contrast sequence for analysis.

### Volumes

The mean volume of measured iodine depositions was not significantly different between the arterial and venous phases in both patient groups (*p* > 0.05; with a residual tumor: arterial volume 12.28 ml ± 3.06, venous volume: 12.25 ml ± 2.99; patients without a residual tumor: arterial volume: 8.44 ml ± 1.85, venous volume: 8.13 ml ± 1.79).

**Fig 3:**
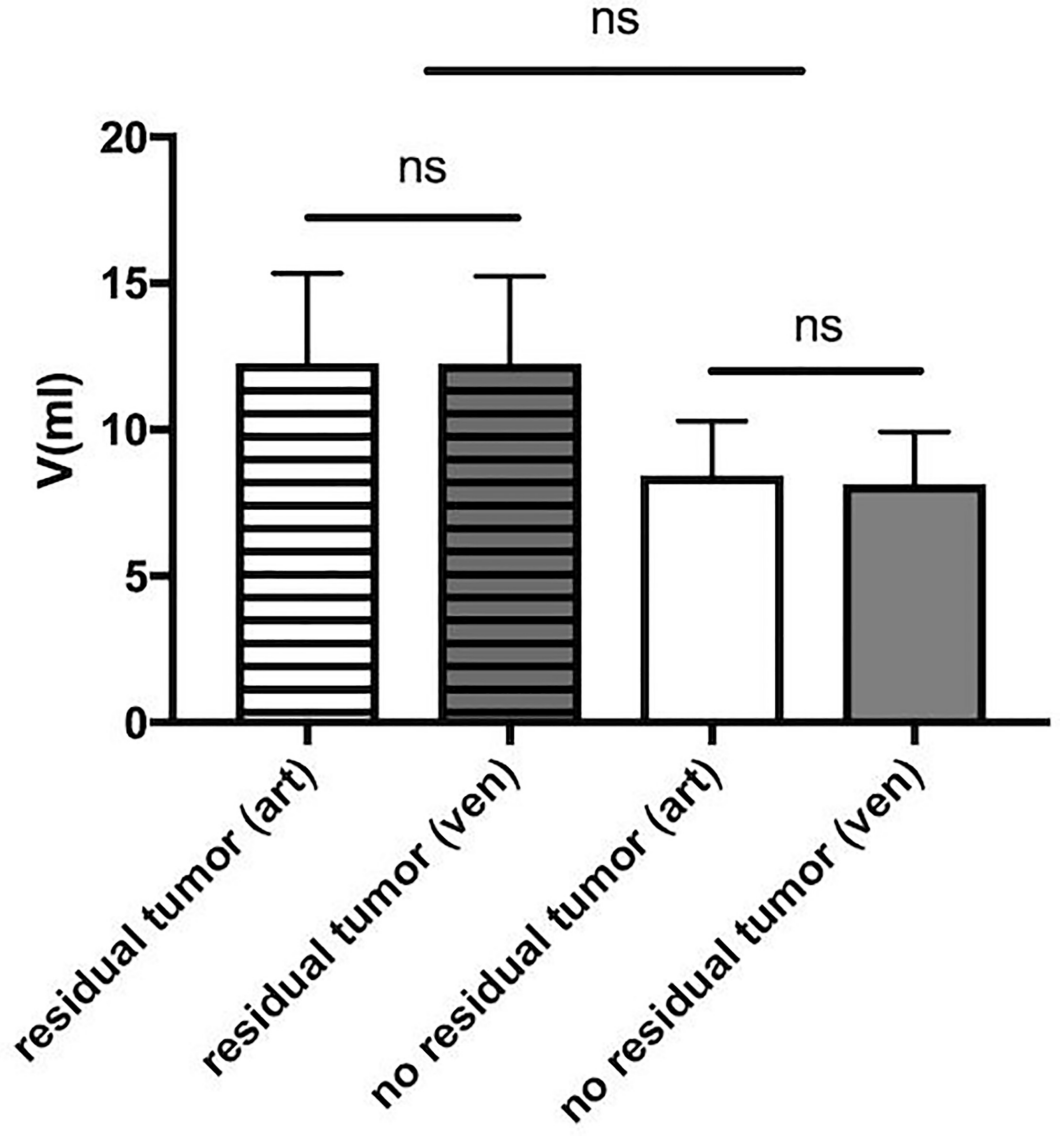
A: Volume measurement of iodine deposition in patients with a (residual tumor) and without a residual tumor (no residual tumor) in the arterial (art) and venous (ven) phases represented as the mean + standard error of the mean (SEM). The volume of iodine depositions did not differ significantly (ns = not significant) between the arterial and venous phases of patients with a residual tumor (arterial volume: 12.28 ml ± 3.06; venous volume: 12.25 ml ± 2.99) and those without a residual tumor (arterial volume: 8.44 ml ± 1.85; venous volume: 8.13 ml ± 1.79).

Furthermore, the mean volume in patients with a residual tumor compared to those without a residual tumor did not differ significantly, as illustrated in Fig 3. The volume of iodine depositions in patients with and without a residual tumor measured in the arterial phase was 12.28 ml ± 3.06, *n* = 14, and 8.44 ± 1.85, *n* = 16 respectively.

**Fig 4:**
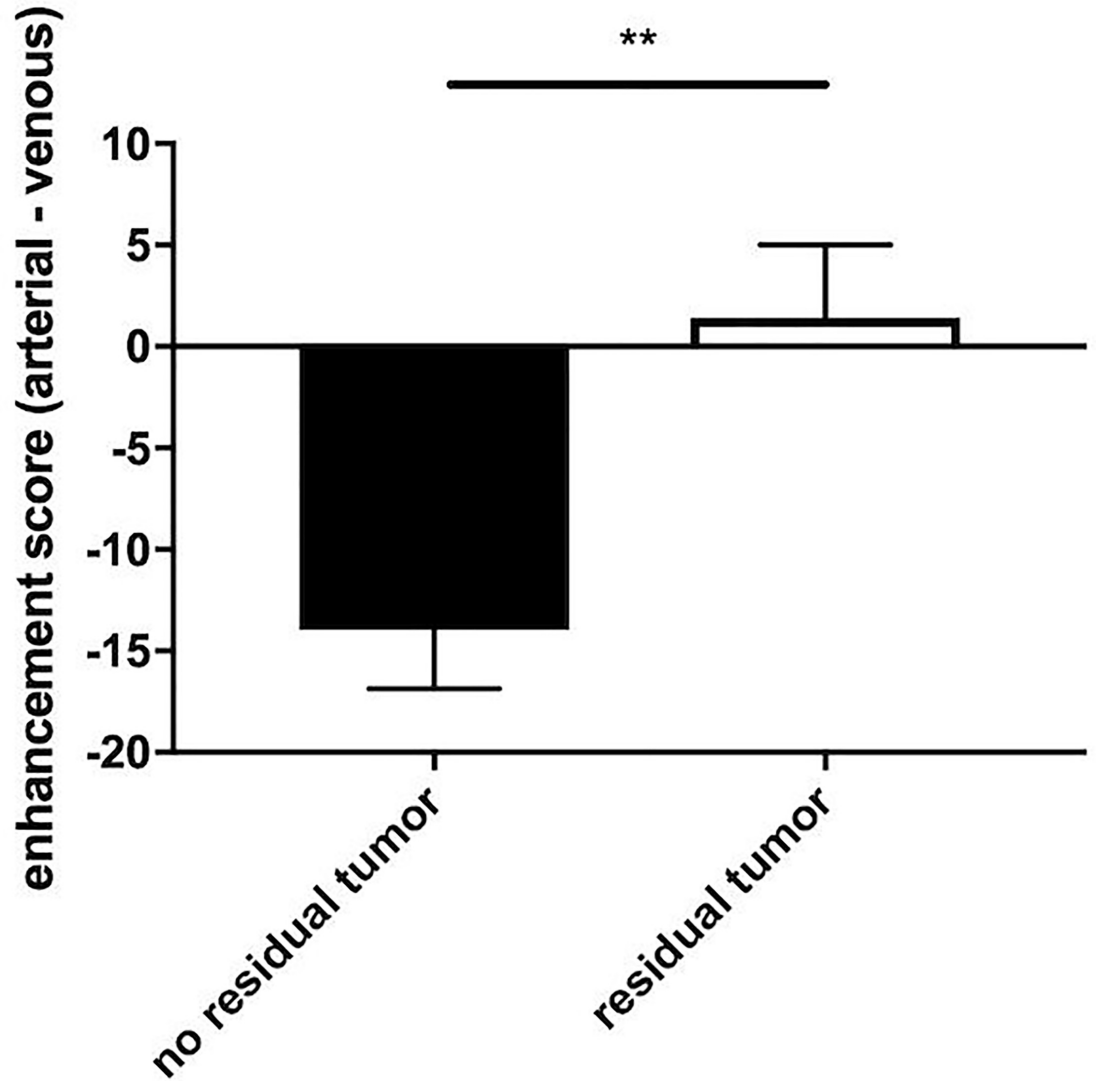
Enhancement scores (mean + SEM) of patients with and without a residual tumor. The enhancement score of patients without a residual tumor (-13.97 ± 2.88) was significantly lower (*p* < 0.01) compared to patients with a residual tumor (1.41 ± 3.59).

The enhancement score was significantly higher in patients with a residual tumor compared to those without a residual tumor, as illustrated in Fig 4 (1.41 ± 3.59, *n* = 14 to -13.97 ± 2.88, *n* = 16; *p* < 0.01). Furthermore, 87.75% of patients with an enhancement score higher than -1 (*n* = 9) had a residual tumor and should be considered for retreatment. In comparison to that, 100.00% of patients with an enhancement score lower than -20.00 (*n* = 6) were shown to be free of tumors.

Furthermore, relative enhancement scores (Fig 2) were significantly lower (*p* < 0.05) in patients without a residual tumor (-0.09 ± 0.02, *n* = 14) in comparison to patients with a residual tumor (0.01 ± 0.02, *n* = 11). Moreover, 83.33% of patients with an enhancement score higher than 0 (*n* = 6) in relation to non-contrast density had a residual tumor and should be considered for retreatment. In comparison to that, 100.00% of patients with an enhancement score lower than -0.13 (*n* = 5) in relation to non-contrast density were shown to be free of tumors.

**Fig 5:**
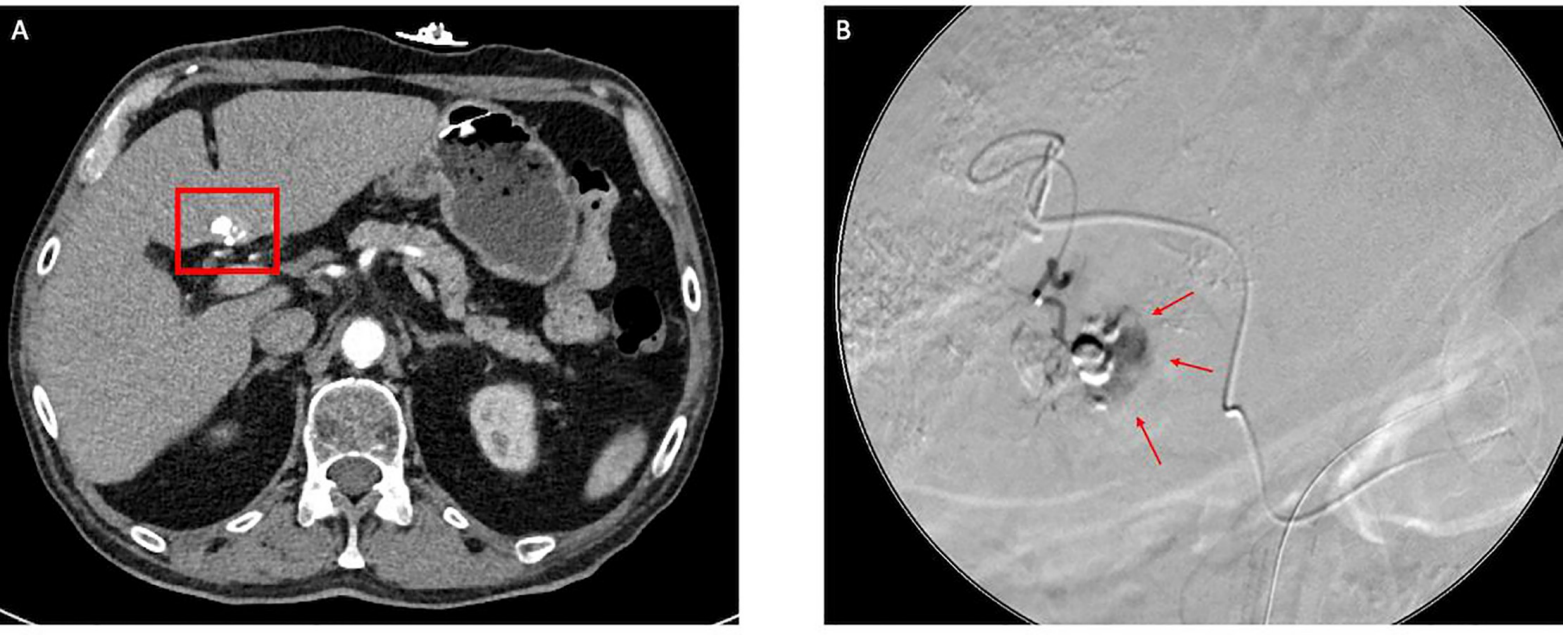
**A: Computed tomography in the arterial phase of a patient with a hidden residual tumor; B: DSA of the same patient unveiling the hidden residual tumor.** Measuring the density of iodine depositions in this patient, an enhancement score of 17.50 clearly revealed a hidden residual tumor beyond the iodine depositions that was diagnosed afterward in the DSA.

From the example in Fig 5, because of density measurements, a hidden residual tumor beyond the iodine depositions could be visualized retrospectively. This tumor was diagnosed later in DSA.

**Fig 6:**
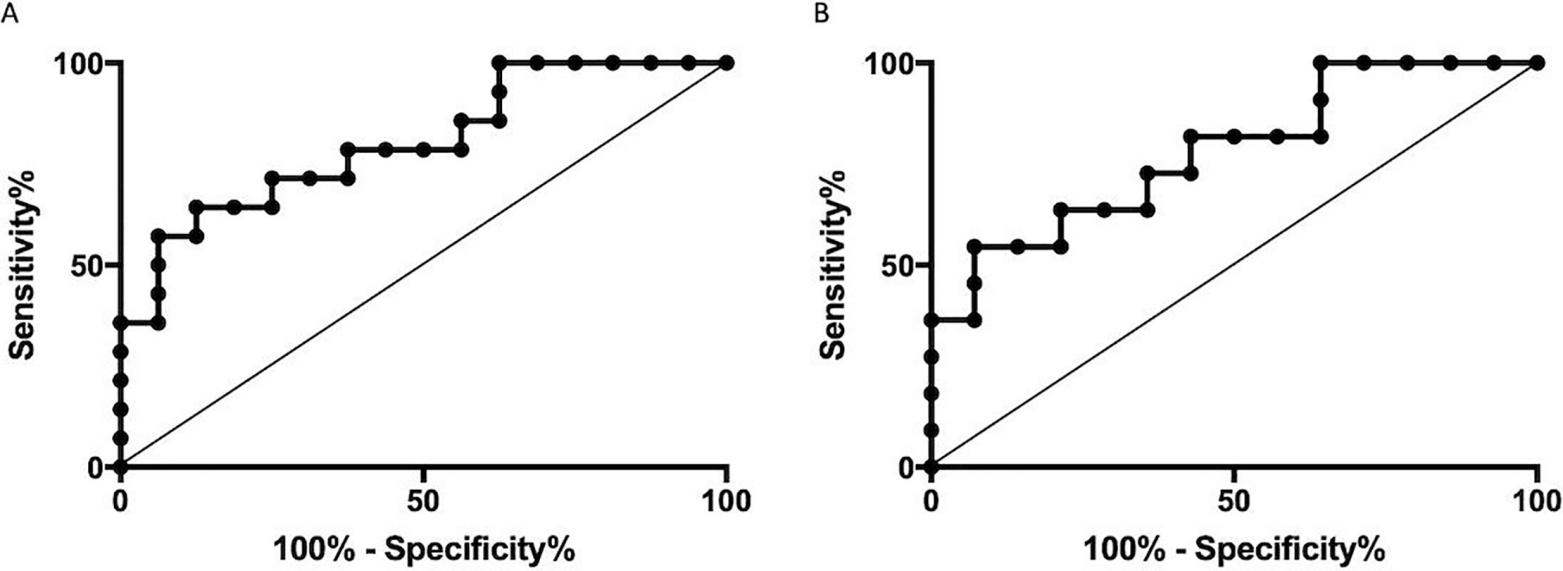
**A: ROC curve of enhancement scores of patients with and without a residual tumor; B: ROC curve of relative enhancement scores (enhancement score / non-contrast density) of patients with and without a residual tumor.** The differentiation between patients with and without a residual tumor was slightly better using enhancement scores (area under the curve = 0.80; *p* < 0.01) rather than relative enhancement scores (area under the curve = 0.79; *p* < 0.05).

Comparing the ROC curves of enhancement scores and relative enhancement scores (Fig 6), a slightly better differentiation between patients with and without a residual tumor was observable in the ROC curve of those scores without relativization on non-contrast densities (area under the curve of enhancement scores = 0.80; area under the curve of relative enhancement scores = 0.79). In our study, two-phasic computed tomographies associated with a lower radiation exposure were not inferior to three-phasic computed tomographies.

## Discussion

In our study, we were able to demonstrate that the density measurements of iodine depositions in the arterial and portal venous phases can unmask the contrast behavior of a hidden residual tumor, and they could therefore help to improve the management of patients after Lipiodol-based cTACE. It was demonstrated that 100.00% of patients with an enhancement score lower than -20.00 (*n* = 6) were shown to be free of tumors. When using our cut-off values, repeated DSAs could have been avoided to exclude a possible residual tumor or tumor relapse after cTACE. Refraining from performing unnecessary DSAs may prevent complications associated with those angiographies, such as bleeding or vascular occlusions [12,13].

The high density of iodinated oil makes it difficult to visually evaluate the contrast behavior of a residual tumor inside the iodine depositions in computed tomographies [10]. We overcame this challenge by measuring the density of iodine depositions in the arterial and venous phases of the CT scan and calculating the difference.

As an alternative, MRI can be used to diagnose an early residual tumor after cTACE with a superior performance in comparison to CT [14]. However, MRI has limitations, since it cannot assess iodine depositions as a marker for the risk of death and progression-free survival [9]. In addition, gadolinium-based contrast agents, which are used in MRI, may deposit in the dentate nucleus and globus pallidus with not-yet-known risks to patient safety [15].

One of the advantages of other TACE techniques, such as DEB-TACE, is the visibility of a residual tumor after TACE because of the non-enhancing nature of the beads [16]. Measuring TACE depositions after cTACE may reduce this technical disadvantage of cTACE in comparison to DEB-TACE.

A limitation of this study was the relatively small number of CTs included (30). To increase the evidence, a prospective multicenter study with a higher patient count would be beneficial to determine the best cut-off values. Furthermore, to increase the confidence in differentiating between patients with and without a residual tumor, further analytics–extracting more imaging features such as radiomics–may be warranted because of the relatively high standard error of the mean of enhancement scores. Another limitation of the study is the use of a 5.00-mm layer thickness when comparing densities in different contrast medium phases. With this relatively thick layer, the tumor can be cut differently in the various contrast medium phases because of patients breathing, which makes the evaluation of small tumors particularly difficult. This limitation can be overcome by using a thinner layer. To make this usable in clinical everyday life, however, automated segmentation is required because of the high workload of segmenting a tumor on thinner layers. Therefore, we believe that, at the time of writing, the use of a 5.00-mm layer thickness is still a method with optimal results, and it can be used in clinical everyday life with adequate effort.

## Conclusion

In conclusion, measuring the enhancement scores of iodine depositions after cTACE helps to exclude an underlying residual tumor after cTACE. As a result, repeated DSAs could be avoided, thus reducing the risk of associated complications. Further prospective investigations are warranted to increase the evidence of our research findings.

## Supporting information

S1 Table Density and volume measurements of patients with and without residual tumor. (XLSX)
